# Effect of antibiotic regimens for bloodstream infections caused by KPC-producing enterobacter: a network meta-analysis

**DOI:** 10.3389/fmed.2026.1743758

**Published:** 2026-06-30

**Authors:** Libo Fei, Huimin Chen, Jia Di, Xiaoyue Zhang, Tan Li, Keqin Liu

**Affiliations:** 1 Department of Emergency Medicine, Jiangsu Second Hospital of Traditional Chinese Medicine (The Second Affiliated Hospital of Nanjing University of Traditional Chinese Medicine), Nanjing, Jiangsu, China; 2 Emergency, Nanjing Pukou People’s Hospital, Liangjiang Hospital Southeast University, Nanjing, Jiangsu, China; 3 Medical Science Liaison, MSD China Holding Co., Ltd., Nanjing, China

**Keywords:** antibiotics, blood stream infections, effect, *Klebsiella pneumoniae*, network meta-analysis

## Abstract

**Introduction:**

This study aimed to assess the impact of different antibiotic regimens on bloodstream infections (BSIs) caused by Klebsiella pneumoniae producing Klebsiella pneumoniae carbapenemase (KPC).

**Methods:**

A thorough literature search was performed using Web of Science, Embase, PubMed, and the Cochrane Library, which yielded 16 studies on antibiotic regimens for KPC-Ent BSI for network meta-analysis (NMA). We calculated pooled overall mortality rates along with 95% confidence intervals (CIs) and utilized the surface under the cumulative ranking curves (SUCRA) to evaluate the efficacy of antibiotic regimens.

**Results:**

Nineteen studies involving 10 treatment regimens were included in the network meta-analysis. For overall mortality, meropenem monotherapy (MEM_Mono) ranked highest, with the greatest probability of being the best treatment (SUCRA 84.0%; PrBest 64.0%). In the subgroup analysis, amikacin monotherapy (AMG_Mono) showed the highest ranking for 28-day mortality (SUCRA 91.3%), whereas MEM_Mono remained the top-ranked regimen for 30-day mortality (SUCRA 83.2%). The consistency and sensitivity analyses supported the robustness of the findings.

**Conclusion:**

Overall, the evidence suggests that MEM_Mono may be the most favorable regimen for KPC-Ent BSI, while the optimal treatment may vary according to the mortality assessment timepoint.

## Introduction

Bloodstream infections (BSI) significantly contribute to mortality, representing an estimated 11–38% of all infection-related deaths (1). Carbapenem-resistant *Enterobacterales* (CRE), especially *Klebsiella pneumoniae* carbapenemase (KPC)-producing *Klebsiella pneumoniae* (Kp), have become a worldwide public health concern owing to their limited treatment options and elevated fatality rates (2–4). In 2020, surveillance data from the European Centre for Disease Prevention and Control (ECDC) indicated a rising incidence of KPC-Kp isolates, reaching 66.3% in Greece, 29.5% in Italy, 48.3% in Romania, and 28.1% in Bulgaria (5). In 2022, carbapenemase-producing Enterobacterales account for 5–30% of BSI cases in endemic regions, with KPC being the most prevalent carbapenemase type. Attributable mortality for KPC-Kp BSI has been reported to range from 30 to 50%, highlighting the critical need for effective therapeutic strategies (6–9). Furthermore, the lack of early and accurate diagnostic methods for carbapenemase detection, along with the unavailability of appropriate antibiotics, exacerbates the mortality (10). The mechanism behind *Klebsiella pneumoniae*’s resistance to carbapenem antibiotics is primarily the production of carbapenemases, which mediate the hydrolysis of cephalosporins, monobactams, carbapenems, and β-lactamase inhibitors (11).

Timely and appropriate antibacterial treatment is crucial for managing patients with BSI (12, 13). Unfortunately, treating BSI patients caused by KPC-Kp presents significant challenges for practitioners. Although current clinical guidelines recommend novel β-lactam/β-lactamase inhibitor combinations as preferred therapy, the evidence base remains heterogeneous and largely derived from observational studies, with moderate to low quality of evidence. A comprehensive synthesis is therefore needed to compare the relative effectiveness of available treatment strategies. This phenomenon primarily stems from the fact that carbapenem resistance frequently co-occurs with co-resistance to multiple essential antibiotic classes, such as fluoroquinolones, β-lactam/β-lactamase inhibitors, and cephalosporins (10, 14–17). Prior to the introduction of novel β-lactam/β-lactamase inhibitor combinations, therapeutic options were largely limited to older antimicrobial agents such as tigecycline (TGC), aminoglycosides, polymyxins, and fosfomycin, with carbapenems occasionally being utilized in certain cases. These options have shown varying efficacy, increasing resistance rates, and significant unacceptable toxicities (18, 19). Additionally, previous retrospective studies on BSI caused by KPC-Kp have found that patients receiving combination antibiotic treatment are significantly more likely to survive than those receiving monotherapy (20–23). Combination therapy involving colistin (COL) plus another antimicrobial agent demonstrating *in vitro* efficacy showed significant mortality reduction, especially in critically ill BSI patients. One reason for this is the low serum levels achievable with TGC (22–25). Although Polymyxin B (PMB) is recognized as a preferred treatment for systemic infections compared to COL, the efficacy of PMB in treating KPC-Kp remains limited to several investigations, which have produced contradictory outcomes regarding the relationship between combination therapy and improved survival (26–29). Emerging evidence indicates that carbapenem-based combination regimens may improve survival outcomes, particularly for infections caused by strains demonstrating *in vitro* susceptibility to these agents (21, 22). Nevertheless, results from multiple studies have demonstrated good clinical outcomes with treatment strategies that do not include carbapenems or aminoglycosides (4, 30, 31).

Previous studies have often reported inconsistent findings regarding BSI caused by KPC-Kp. Moreover, there are few studies that have directly compared outcomes from the different antibiotic regimens. In such cases, network meta-analysis (NMA) offers a valuable approach for the simultaneous synthesis of all available evidence. This study aimed to verify the effect of available treatment regimens on KPC-induced BSI via NMA.

## Materials and methods

This research was conducted in accordance with the Preferred Reporting Items for Systematic Reviews and Meta-Analyses (PRISMA) guidelines, ensuring methodological rigor and transparent reporting throughout our investigation (32).

### Database search

PubMed, Embase, Web of Science, and the Cochrane Library were independently searched by two authors. All databases were searched up to March 26, 2026, with studies limited to those published in English. The systematic literature search employed the following key terms and Medical Subject Headings (MeSH): Carbapenem Antibiotics, Carbapenems, Carbapenem resistan*, Klebsiella pneumoniae aerogenes, Klebsiella pneumoniae, Bloodstream Infections, Pyaemia, Septicemia, Blood Poisoning, Citrobacter freundii, KPC, Klebsiella pneumoniae carbapenemase, Enterobacter hormaechei, Enterobacterales and Sepsis. The complete search syntax, including database-specific adaptations, is provided in Supplementary Table S1. Inclusion criteria were: (1) Population: participants with confirmed BSI caused by KPC-producing Enterobacter; (2) Intervention and comparison: antibiotics of any kind; (3) Outcomes: mortality; (4) Study design: non-interventional studies or randomized controlled trials (RCTs). Exclusion criteria were: (1) studies from which data could not be extracted or transformed; (2) case reports, reviews, communications, animal studies, and editorials; (3) non-English publications. Two investigators independently evaluated article titles and abstracts, with final inclusion determinations made after full-text review. Any discrepancies were resolved through consensus discussion involving a third researcher.

### Data extraction and risk of bias assessment

Two reviewers independently extracted the following data elements from all eligible studies using an electronic form: publication year, author, country, study design, sample size, proportion of male participants, age, diagnosis methods, therapies, and clinical outcomes on mortality. Disagreements were resolved through discussion; when consensus could not be reached, a third author (Keqin Liu) was consulted. The classification of antibiotic regimens was based on Aminoglycoside Monotherapy (AMG_Mono), Ceftazidime-avibactam (CAZ_AVI), Carbapenem-based Combination Therapy (CBP_Comb), Carbapenem Monotherapy (CBP_Mono), Meropenem-based Combination Therapy (MEM_Comb), Meropenem Monotherapy (MEM_Mono), Other Regimens (Others), Polymyxin-based Combination Therapy (POL_Comb), Polymyxin Monotherapy (POL_Mono), and Tigecycline Monotherapy (TGC_Mono). Monotherapy categories (AMG_Mono, CBP_Mono, MEM_Mono, POL_Mono, and TGC_Mono) were defined as regimens that did not include any additional concomitant antibacterial agents, particularly excluding combinations with CAZ-AVI, carbapenems, polymyxins, tigecycline, or aminoglycosides. When available, adjusted effect estimates were preferentially extracted; otherwise, unadjusted event data were used.

For the assessment of risk of bias in RCTs, the Cochrane Collaboration’s tool was employed (33). The methodological rigor of the included studies was systematically appraised across five critical domains: selection, performance, detection, attrition, and reporting. Each domain received a categorical assessment indicating either low, moderate, or uncertain risk of methodological bias (33). For non-randomized controlled trials, methodological quality was assessed using the Newcastle-Ottawa Scale (NOS), which examines three key domains: (1) cohort selection (including representativeness, control group selection, and baseline outcome status), (2) comparability between groups (based on study design and analysis), and (3) outcome evaluation (assessment method and follow-up adequacy). Studies were stratified by total NOS score (9-point scale) into three bias risk categories: high (0–3), moderate (4–6), and low (7–9) (34). Two reviewers independently evaluated the methodological quality of each included study, with inter-rater discrepancies reconciled through consensus-based discussion to ensure consistent bias assessment.

### Statistical analysis

Statistical analyses were conducted using Stata 18.0 (StataCorp) and R 4.1.2 (R Foundation). The network meta-analysis employed a frequentist approach with random-effects assumptions. The design-by-treatment interaction random effects model assessed global consistency and inconsistency (35). When an inconsistency factor exceeding 2 was identified, the characteristics of studies within affected loops were explored, followed by a sensitivity analysis that excluded these studies. Local consistency and inconsistency were evaluated separately using the network node-splitting model to examine the transitivity assumption for this network meta-analysis (36), in this approach, a *p* > 0.05 indicated consistency between direct and indirect evidence, whereas *p* ≤ 0.05 suggested potential inconsistency requiring further investigation. To investigate potential sources of heterogeneity, we conducted pre-specified subgroup analyses based on clinical and methodological characteristics. The robustness of pooled estimates was verified through leave-one-out sensitivity analysis, systematically excluding individual studies to assess their influence on overall results (37). Treatment hierarchies were established using surface under the cumulative ranking (SUCRA) probabilities, with higher values (0–100%) indicating greater likelihood of being the optimal therapeutic approach for bloodstream infections (38). The SUCRA metric provides a probabilistic ranking of interventions, where higher SUCRA values indicate better efficacy, whereas lower values suggest poorer performance. Pairwise treatment effects were summarized in a league table (ORs with 95% CIs). Statistical significance was defined as *p* < 0.05 (two-tailed). Finally, funnel plot symmetry assessment revealed no significant publication bias.

## Results

### Overview of studies

**FIGURE 1:**
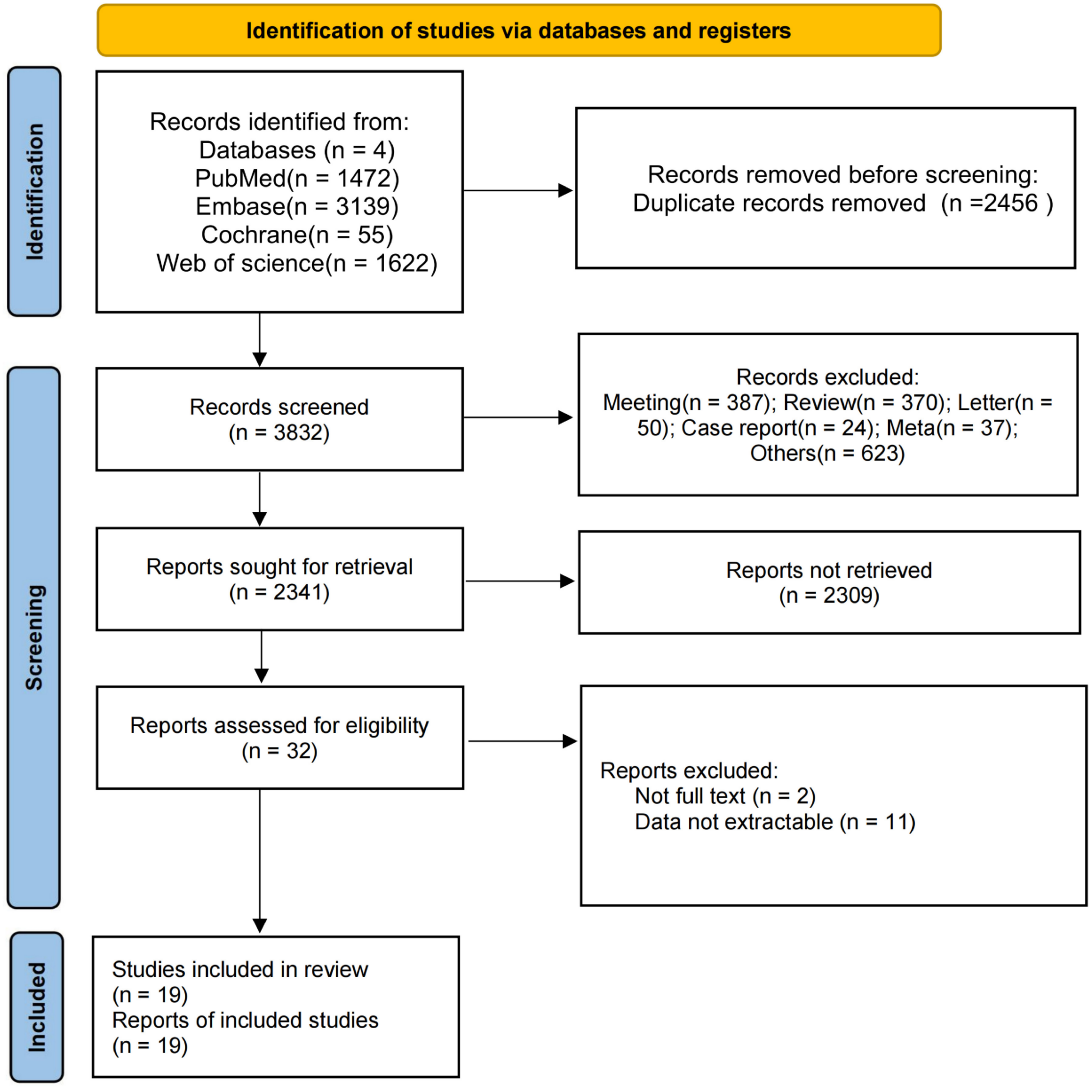
Flowchart of the literature search.

A total of 6,288 citations were obtained from the database search. Following screening titles and abstracts, 2,456 duplicate citations and 3,800 irrelevant studies were excluded. After comprehensive evaluation of the 32 eligible full-text articles, 19 studies met the predefined inclusion criteria and were incorporated into the NMA (21–23, 39–54) (Figure 1). The 19 eligible studies spanned an 14-year publication period (2012–2026), comprising 18 retrospective cohort analyses (94.7%) and one prospective cross-sectional investigation (5.3%) (Table 1).

**TABLE 1:** Study characteristics.

Author	Year	Study design	Country	Number of patients	Age, y	Male, %	Presentation with sepsis or septic shock, %	Diagnosis	Treatment regimens	Outcomes, n (%)
Qureshi	2012	Retrospective cohort	USA	34	NS	44.1	NS	Blood cultures	Polymyxin-based combination	28-day mortality 5 (35.7%)
									Carbapenem-based combination	28-day mortality 1 (25%)
									others	28-day mortality 1 (16.7%)
									Tigecycline monotherapy	28-day mortality 4 (80%)
									Carbapenems monotherapy	28-day mortality 2 (50%)
									Aminoglycoside monotherapy	28-day mortality 0 (0%)
Tumbarello	2012	Multicenter retrospective cohort study	Italy	125	62.3 ± 15.6	58.4	13.6	Blood cultures	Tigecycline monotherapy	30-day mortality1 0 (52.6%)
									Polymyxin monotherapy	30-day mortality1 1 (50%)
									Aminoglycoside monotherapy	30-day mortality 4 (80%)
									Polymyxin-based combination	30-day mortality 9 (23.1%)
									Others	30-day mortality 6 (50%)
Daikos	2014	retrospective observational study	Greece	205	62.7 ± 17.5	57.6	67.3	Blood cultures	Carbapenem-based combination	28-day mortality 3 (12.5%)
									Polymyxin-based combination	28-day mortality1 6 (28.6%)
									Others	28-day mortality 9 (45%)
									Tigecycline monotherapy	28-day mortality1 1 (40.7%)
									Polymyxin monotherapy	28-day mortality1 2 (54.5%)
									Aminoglycoside monotherapy	28-day mortality 2 (22.2%)
									Carbapenems monotherapy	28-day mortality 7 (58.3%)
Gonzalez-Padilla	2014	Retrospective cohort	Spain	50	60.5 (19–86)	64	60	Clinical evidence of infection with criteria for sepsis	Tigecycline monotherapy	30-day mortality 8 (88.9%)
									Aminoglycoside monotherapy	30-day mortality 8 (100%)
									Meropenem monotherapy	30-day mortality 0 (0%)
									Others	30-day mortality2 1 (100%)
									Meropenem-based combination	30-day mortality 0 (0%)
									Polymyxin-based combination	30-day mortality 0 (0%)
Gomez-Simmonds	2016	Retrospective observational study	USA	141	NS	61	30.5	Blood cultures	Polymyxin monotherapy	30-day mortality 7 (28%)
									Tigecycline monotherapy	30-day mortality 7 (26.9%)
									Aminoglycoside monotherapy	30-day mortality 2 (14.3%)
									Polymyxin-based combination	30-day mortality5 4 (50.9%)
									Others	30-day mortality 7 (36.8%)
Shields	2017	Retrospective observational study	USA	68	NS	60.3	NS	Blood cultures	Ceftazidime/avibactam	30-day mortality 1 (7.7%)
									Carbapenem-based combination	30-day mortality 8 (32%)
									Polymyxin-based combination	30-day mortality 9 (30%)
Medeiros	2019	Retrospective cohort	Brazil		57.6 ± 17.0	64.6	NS	Blood cultures	Polymyxin monotherapy	30-day mortality 4 (50%)
									Meropenem monotherapy	30-day mortality 1 (100%)
									Aminoglycoside monotherapy	30-day mortality 1 (50%)
									Polymyxin-based combination	30-day mortality3 4 (47.9%)
Falcone	2020	Retrospective observational study	Italy	74	NS	NS	39.2	Blood cultures	Ceftazidime/avibactam	30-day mortality 3 (23.1%)
									Polymyxin-based combination	30-day mortality2 7 (44.3%)
Papadimitriou-Olivgeris	2020	Retrospective observational study	Greece	70	56.0 ± 18.4	68.6	58.6	Blood cultures	Tigecycline monotherapy	30-day mortality1 5 (21.4%)
Aslan	2022	Retrospective cohort	Turkey	124	62.1 ± 18.0	53.2	52.4	Blood cultures	Carbapenems monotherapy	30-day mortality 6 (33.3%)
									Polymyxin monotherapy	30-day mortality 1 (25%)
									Others	30-day mortality 1 (33.3%)
									Polymyxin-based combination	30-day mortality 2 (28.6%)
									Meropenem-based combination	30-day mortality 0 (0%)
Fang	2023	Retrospective cohort	China	184	62.0 ± 15.5	71.2	NS	Blood cultures	Others	30-day mortality3 2 (30.8%)
									Tigecycline monotherapy	30-day mortality1 4 (33.3%)
									Aminoglycoside monotherapy	30-day mortality 2 (13.3%)
									Polymyxin monotherapy	30-day mortality 6 (40%)
									Ceftazidime/avibactam	30-day mortality 6 (19.4%)
									Carbapenems monotherapy	30-day mortality4 4 (40%)
Caston	2022	Multicenter retrospective cohort study	Spain	339	67(56–77)	66.1	NS	Blood cultures	Ceftazidime/avibactam	30-day mortality2 6 (13.8%)
									Polymyxin monotherapy	30-day mortality3 3 (22%)
Crooker	2026	Retrospective cohort	USA	112	57.1(16.3)	52	NS	Blood cultures	Ceftazidime/avibactam	30-day mortality 9 (21.4%)
									Meropenem-based combination	30-day mortality2 6 (37.1%)
Huang	2025	Retrospective cohort	China	101	71(20.5)	51.06	NS	Blood cultures	Ceftazidime/avibactam	28-day mortality1 3 (27.7%)
									Carbapenem-based combination	28-day mortality1 8 (33.3%)

NS, not specified.

### Study quality assessment

The risk of bias for each included study was rated. 18 out of 19 studies were evaluated as having a low risk of bias, and one was assessed to be at moderate risk (Table 2).

**TABLE 2:** Quality evaluation of included studies.

Study	Representa- tiveness of the exposed cohort	Selection of the non-exposed cohort	Ascertainment of exposure	Demonstration that the outcome of interest was not present at the start of the study	Comparability of cohorts on the basis of the design or analysis	Assessment of outcome	Was follow-up long enough for outcomes to occur	Adequacy of follow up of cohorts	Total quality scores
Qureshi et al. (21)	1	1	1	1	1	1	1	1	8
Tumbarello et al. (22)	1	1	1	1	1	1	1	1	8
Daikos et al. (23)	1	1	1	1	1	1	1	1	8
Gonzalez-Padilla et al. (63)	1	1	1	1	1	1	1	1	8
Gomez-Simmonds et al. (39)	1	1	1	1	1	1	1	1	8
Shields et al. (40)	1	1	1	1	1	1	1	1	8
Jahidul Hasan et al. (41)	1	1	1	1	1	1	0	0	6
Liang et al. (42)	1	1	1	1	1	1	1	1	8
Medeiros et al. (43)	1	1	1	1	1	1	1	1	8
Falcone et al. (44)	1	1	1	1	1	1	1	1	8
Papadimitriou-Olivgeris et al. (45)	1	1	1	1	1	1	1	1	8
Lee et al. (46)	1	1	1	1	1	1	1	1	8
Shen et al. (47)	1	1	1	1	1	1	1	1	8
Aslan et al. (48)	1	1	1	1	1	1	1	1	8
Lima et al. (49)	1	1	1	1	1	1	1	1	8
Fang et al. (50)	1	1	1	1	1	1	1	1	8
Castón et al. (52)	1	1	1	1	1	1	1	1	8
Crooker et al. (53)	1	1	1	1	1	1	1	1	8
Huang et al. (54)	1	1	1	1	1	1	1	1	8

The maximum score on the NOS is 9 (highest quality), and we assigned scores of 0–3, 4–6, and 7–9 for low, moderate, and high quality of studies, respectively.

### Network meta-analysis

#### All-cause mortality

**FIGURE 2:**
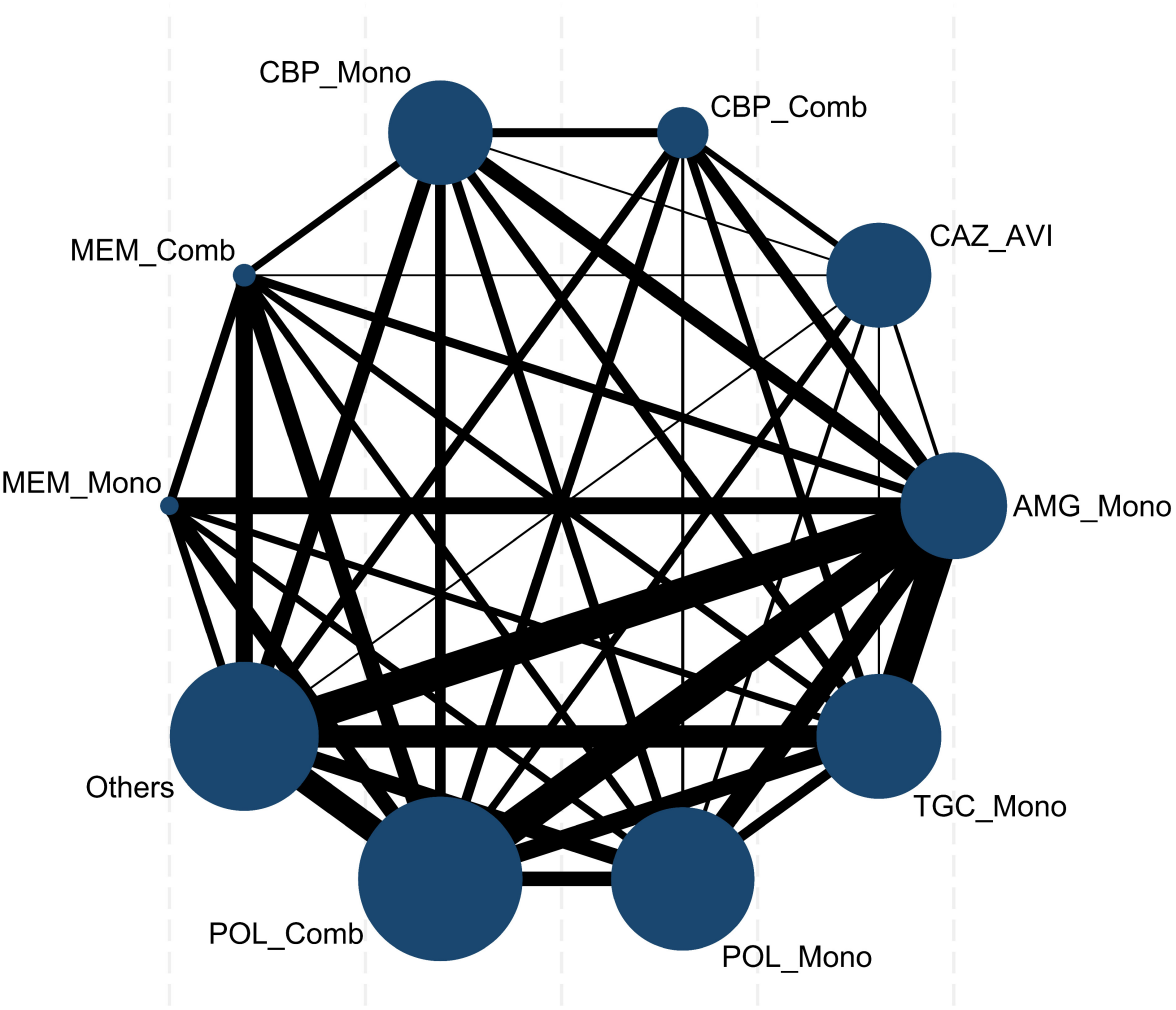
Network plot for the analysis of overall mortality.

**FIGURE 3:**
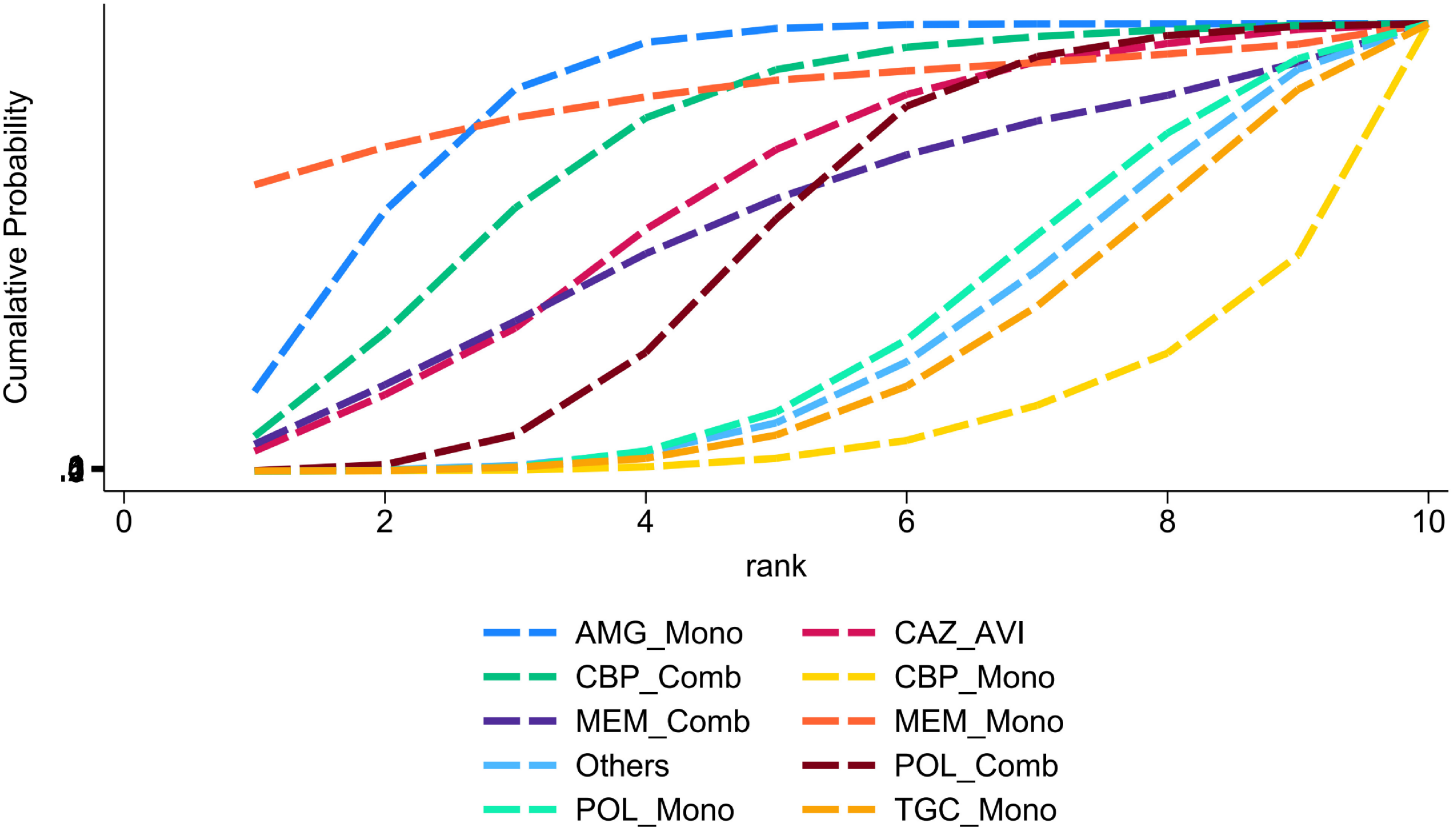
Cumulative probabilities of treatment effect on overall mortality.

The consistency test indicated that the consistency model was more appropriate. The analysis included 19 studies with data on 10 treatment regimens related to overall mortality. The treatment regimens assessed were AMG_Mono, CAZ_AVI, CBP_Comb, CBP_Mono, MEM_Comb, MEM_Mono, Others, POL_Comb, POL_Mono, and TGC_Mono (Figure 2). The SUCRA analysis indicated that MEM_Mono (84.0%) was the most likely effective treatments for BSI caused by KPC-Ent (Figure 3). Among these, MEM_Mono had the highest probability of being the best treatment (PrBest 64.0%). In terms of pairwise comparisons, MEM_Mono showed a non-significant trend toward lower mortality compared with CAZ_AVI (OR = 0.46 [–0.58, 1.50]) and did not significantly differ from CBP_Comb (OR = 0.30 [–1.12, 1.72]). MEM_Mono was significantly superior, CBP_Mono (OR = 1.11 [0.09, 2.12]), POL_Mono (OR = 0.96 [0.23, 1.70]), and TGC_Mono (OR = 1.06 [0.21, 1.91]). Additionally, CAZ_AVI was associated with significantly lower mortality compared with CBP_Mono (OR = –0.86 [–1.87, –0.16]), and CBP_Comb significantly reduced mortality compared with CBP_Mono (OR = –1.11 [–2.12, –0.09]). Although POL_Comb showed a trend toward improved outcomes over POL_Mono, the difference was not statistically significant (OR = –0.32 [–0.94, 0.31]) (Table 3).

**TABLE 3:** Network meta-analysis of the overall mortality of different treatment regimens.

TGC_Mono	−0.10 (−0.80, 0.61)	−0.41 (−1.09, 0.26)	−0.05 (−0.74, 0.65)	−1.63 (−3.99, 0.73)	−0.54 (−1.94, 0.85)	0.26 (−0.54, 1.07)	−0.84 (−1.80, 0.12)	−0.60 (−1.52, 0.33)	−1.06 (−1.91, −0.21)
0.10 (−0.61, 0.80)	**POL_Mono**	−0.32 (−0.94, 0.31)	0.05 (−0.65, 0.75)	−1.53 (−3.90, 0.83)	−0.45 (−1.79, 0.89)	0.36 (−0.44, 1.15)	−0.75 (−1.66, 0.17)	−0.50 (−1.42, 0.42)	−0.96 (−1.70, −0.23)
0.41 (−0.26, 1.09)	0.32 (−0.31, 0.94)	**POL_Comb**	0.37 (−0.31, 1.04)	−1.21 (−3.55, 1.12)	−0.13 (−1.45, 1.19)	0.68 (−0.10, 1.45)	−0.43 (−1.27, 0.41)	−0.18 (−1.08, 0.71)	−0.65 (−1.38, 0.09)
0.05 (−0.65, 0.74)	−0.05 (−0.75, 0.65)	−0.37 (−1.04, 0.31)	**Others**	−1.58 (−3.95, 0.79)	−0.50 (−1.88, 0.89)	0.31 (−0.47, 1.09)	−0.80 (−1.75, 0.16)	−0.55 (−1.48, 0.38)	−1.01 (−1.85, −0.18)
1.63 (−0.73, 3.99)	1.53 (−0.83, 3.90)	1.21 (−1.12, 3.55)	1.58 (−0.79, 3.95)	**MEM_Mono**	1.08 (−1.53, 3.70)	1.89 (−0.52, 4.30)	0.78 (−1.67, 3.23)	1.03 (−1.39, 3.46)	0.57 (−1.84, 2.97)
0.54 (−0.85, 1.94)	0.45 (−0.89, 1.79)	0.13 (−1.19, 1.45)	0.50 (−0.89, 1.88)	−1.08 (−3.70, 1.53)	**MEM_Comb**	0.81 (−0.61, 2.22)	−0.30 (−1.72, 1.12)	−0.05 (−1.57, 1.47)	−0.52 (−1.70, 0.66)
-0.26 (−1.07, 0.54)	−0.36 (−1.15, 0.44)	−0.68 (−1.45, 0.10)	−0.31 (−1.09, 0.47)	−1.89 (−4.30, 0.52)	−0.81 (−2.22, 0.61)	**CBP_Mono**	−1.11 (−2.12, −0.09)	−0.86 (−1.87, 0.16)	−1.32 (−2.21, −0.43)
0.84 (−0.12, 1.80)	0.75 (−0.17, 1.66)	0.43 (−0.41, 1.27)	0.80 (−0.16, 1.75)	−0.78 (−3.23, 1.67)	0.30 (−1.12, 1.72)	1.11 (0.09, 2.12)	**CBP_Comb**	0.25 (−0.88, 1.38)	−0.22 (−1.08, 0.65)
0.60 (−0.33, 1.52)	0.50 (−0.42, 1.42)	0.18 (−0.71, 1.08)	0.55 (−0.38, 1.48)	−1.03 (−3.46, 1.39)	0.05 (−1.47, 1.57)	0.86 (−0.16, 1.87)	−0.25 (−1.38, 0.88)	**CAZ_AVI**	−0.46 (−1.50, 0.58)
1.06 (0.21, 1.91)	0.96 (0.23, 1.70)	0.65 (−0.09, 1.38)	1.01 (0.18, 1.85)	−0.57 (−2.97, 1.84)	0.52 (−0.66, 1.70)	1.32 (0.43, 2.21)	0.22 (−0.65, 1.08)	0.46 (−0.58, 1.50)	**AMG_Mono**

Estimates are presented as mean differences with 95% CI in parentheses. Mean differences below 1 favor the column intervention and mean differences above 1 favor the row intervention. Interventions which are significantly different since the 95% CI below 1. TGC_Mono, tigecycline monotherapy; POL_Mono, polymyxin monotherapy; POL_Comb, polymyxin-based combination therapy; Others, other regimens; MEM_Mono, meropenem monotherapy; MEM_Comb, meropenem-based combination therapy; CBP_Mono, carbapenem monotherapy; CBP_Comb, carbapenem-based combination therapy; CAZ_AVI, ceftazidime-avibactam; AMG_Mono, aminoglycoside monotherapy.

#### Inconsistency analysis

The loop-specific inconsistency assessment revealed no statistically significant disagreement within any closed loops of the treatment network (all *P* = 0.968, Supplementary Figures S1). The node-splitting analysis demonstrated good agreement between direct and indirect evidence across all treatment comparisons, with no statistically significant discrepancies observed (Supplementary Table S2). Thus, the consistency assessment results confirmed the reliability of the NMA findings.

#### Subgroup analysis

**FIGURE 4:**
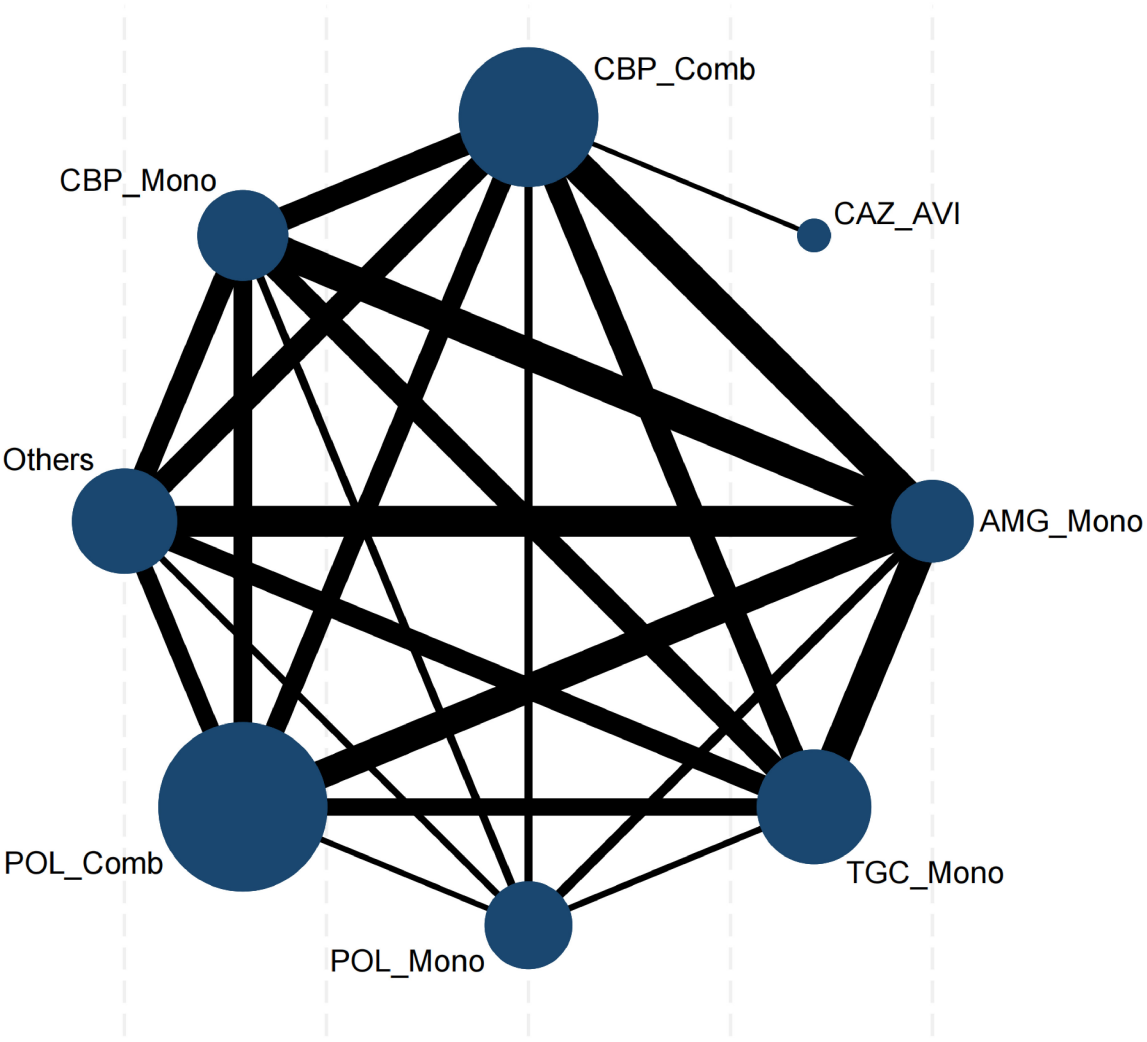
Network plot for the analysis of 28-day mortality.

**FIGURE 5:**
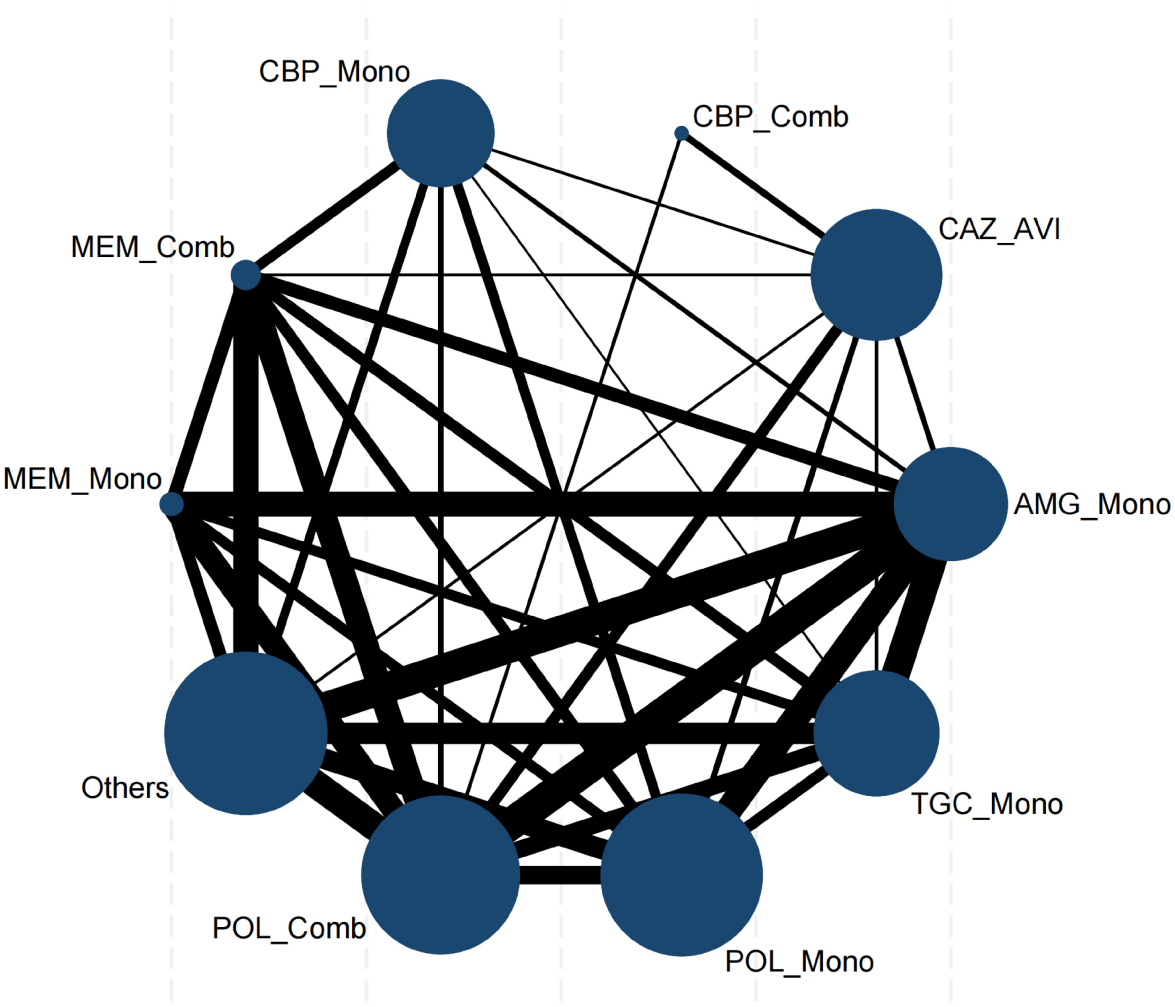
Network plot for the analysis of 30-day mortality.

Subgroup analyses were stratified by mortality assessment timepoints (e.g., 28-day and 30-day mortality). Three studies reported outcomes on 28-day mortality involving eight regimens, while 11 studies reported on 30-day mortality with ten regimens (Figures 4, 5). The SUCRA analysis indicated that AMG_Mono (91.3%) was the most likely effective treatments for BSI in 28-day (Table 4 and Supplementary Figure S2). Regarding 30-day mortality, MEM_Mono (83.2%) was the most likely effective treatments (Table 5 and Supplementary Figure S3).

**TABLE 4:** Network meta-analysis of the 28 day-mortality of different treatment regimens.

POL_Comb	0.38 (−0.72, 1.49)	−0.70 (−1.57, 0.18)	−0.27 (−1.33, 0.80)	0.35 (−0.85, 1.55)	−1.52 (−2.76, −0.27)	−1.08 (−2.66, 0.50)	−1.78 (−3.30, −0.27)
-0.38 (−1.49, 0.72)	**Others**	−1.08 (−2.08, −0.08)	−0.65 (−1.81, 0.51)	−0.04 (−1.33, 1.26)	−1.90 (−3.24, −0.56)	−1.46 (−3.11, 0.19)	−2.17 (−3.76, −0.58)
0.70 (−0.18, 1.57)	1.08 (0.08, 2.08)	**MEM_Mono**	0.43 (−0.51, 1.37)	1.05 (−0.04, 2.13)	−0.82 (−1.96, 0.32)	−0.38 (−1.88, 1.12)	−1.09 (−2.51, 0.34)
0.27 (−0.80, 1.33)	0.65 (−0.51, 1.81)	−0.43 (−1.37, 0.51)	**MEM_Comb**	0.62 (−0.63, 1.86)	−1.25 (−2.54, 0.05)	−0.81 (−2.43, 0.81)	−1.52 (−3.07, 0.04)
-0.35 (−1.55, 0.85)	0.04 (−1.26, 1.33)	−1.05 (−2.13, 0.04)	−0.62 (−1.86, 0.63)	**CBP_Mono**	−1.86 (−3.27, −0.46)	−1.43 (−3.14, 0.28)	−2.13 (−3.77, −0.49)
1.52 (0.27, 2.76)	1.90 (0.56, 3.24)	0.82 (−0.32, 1.96)	1.25 (−0.05, 2.54)	1.86 (0.46, 3.27)	**CBP_Comb**	0.44 (−1.31, 2.18)	−0.27 (−1.12, 0.59)
1.08 (−0.50, 2.66)	1.46 (−0.19, 3.11)	0.38 (−1.12, 1.88)	0.81 (−0.81, 2.43)	1.43 (−0.28, 3.14)	−0.44 (−2.18, 1.31)	**CAZ_AVI**	−0.70 (−2.65, 1.24)
1.78 (0.27, 3.30)	2.17 (0.58, 3.76)	1.09 (−0.34, 2.51)	1.52 (−0.04, 3.07)	2.13 (0.49, 3.77)	0.27 (−0.59, 1.12)	0.70 (−1.24, 2.65)	**AMG_Mono**

Estimates are presented as mean differences with 95% CI in parentheses. Mean differences below 1 favor the column intervention and mean differences above 1 favor the row intervention. TGC_Mono, tigecycline monotherapy; POL_Mono, polymyxin monotherapy; POL_Comb, polymyxin-based combination therapy; Others, other regimens; MEM_Mono, meropenem monotherapy; MEM_Comb, meropenem-based combination therapy; CBP_Mono, carbapenem monotherapy; CBP_Comb, carbapenem-based combination therapy; CAZ_AVI, ceftazidime-avibactam; AMG_Mono, aminoglycoside monotherapy.

**TABLE 5:** Network meta-analysis of the 30 day-mortality of different treatment regimens.

TGC_Mono	−0.15 (−1.06, 0.76)	−0.26 (−1.20, 0.67)	0.11 (−0.81, 1.03)	−1.53 (−3.97, 0.91)	−0.55 (−2.16, 1.06)	0.20 (−0.92, 1.33)	0.03 (−1.80, 1.85)	−0.36 (−1.51, 0.80)	−1.01 (−2.08, 0.05)
0.15 (−0.76, 1.06)	**POL_Mono**	−0.11 (−0.93, 0.70)	0.26 (−0.65, 1.16)	−1.38 (−3.81, 1.05)	−0.40 (−1.92, 1.12)	0.35 (−0.72, 1.42)	0.17 (−1.59, 1.94)	−0.21 (−1.34, 0.93)	−0.87 (−1.76, 0.03)
0.26 (−0.67, 1.20)	0.11 (−0.70, 0.93)	**POL_Comb**	0.37 (−0.58, 1.33)	−1.27 (−3.66, 1.13)	−0.29 (−1.79, 1.22)	0.47 (−0.63, 1.57)	0.29 (−1.33, 1.91)	−0.09 (−1.24, 1.05)	−0.75 (−1.67, 0.17)
-0.11 (−1.03, 0.81)	−0.26 (−1.16, 0.65)	−0.37 (−1.33, 0.58)	**Others**	−1.64 (−4.10, 0.82)	−0.66 (−2.29, 0.97)	0.09 (−0.99, 1.18)	−0.08 (−1.91, 1.75)	−0.47 (−1.62, 0.69)	−1.12 (−2.20, −0.05)
1.53 (−0.91, 3.97)	1.38 (−1.05, 3.81)	1.27 (−1.13, 3.66)	1.64 (−0.82, 4.10)	**MEM_Mono**	0.98 (−1.74, 3.70)	1.73 (−0.81, 4.28)	1.55 (−1.32, 4.43)	1.17 (−1.35, 3.69)	0.51 (−1.97, 3.00)
0.55 (−1.06, 2.16)	0.40 (−1.12, 1.92)	0.29 (−1.22, 1.79)	0.66 (−0.97, 2.29)	−0.98 (−3.70, 1.74)	**MEM_Comb**	0.75 (−0.91, 2.41)	0.57 (−1.58, 2.73)	0.19 (−1.57, 1.96)	−0.47 (−1.80, 0.87)
-0.20 (−1.33, 0.92)	−0.35 (−1.42, 0.72)	−0.47 (−1.57, 0.63)	−0.09 (−1.18, 0.99)	−1.73 (−4.28, 0.81)	−0.75 (−2.41, 0.91)	**CBP_Mono**	−0.18 (−2.09, 1.74)	−0.56 (−1.89, 0.77)	−1.22 (−2.38, −0.05)
-0.03 (−1.85, 1.80)	−0.17 (−1.94, 1.59)	−0.29 (−1.91, 1.33)	0.08 (−1.75, 1.91)	−1.55 (−4.43, 1.32)	−0.57 (−2.73, 1.58)	0.18 (−1.74, 2.09)	**CBP_Comb**	−0.38 (−2.32, 1.56)	−1.04 (−2.80, 0.72)
0.36 (−0.80, 1.51)	0.21 (−0.93, 1.34)	0.09 (−1.05, 1.24)	0.47 (−0.69, 1.62)	−1.17 (−3.69, 1.35)	−0.19 (−1.96, 1.57)	0.56 (−0.77, 1.89)	0.38 (−1.56, 2.32)	**CAZ_AVI**	−0.66 (−1.93, 0.62)
1.01 (−0.05, 2.08)	0.87 (−0.03, 1.76)	0.75 (−0.17, 1.67)	1.12 (0.05, 2.20)	−0.51 (−3.00, 1.97)	0.47 (−0.87, 1.80)	1.22 (0.05, 2.38)	1.04 (−0.72, 2.80)	0.66 (−0.62, 1.93)	**AMG_Mono**

Estimates are presented as mean differences with 95% CI in parentheses. Mean differences below 1 favor the column intervention and mean differences above 1 favor the row intervention. Interventions which are significantly different since the 95% CI below 1. TGC_Mono, tigecycline monotherapy; POL_Mono, polymyxin monotherapy; POL_Comb, polymyxin-based combination therapy; Others, other regimens; MEM_Mono, meropenem monotherapy; MEM_Comb, meropenem-based combination therapy; CBP_Mono, carbapenem monotherapy; CBP_Comb, carbapenem-based combination therapy; CAZ_AVI, ceftazidime-avibactam; AMG_Mono, aminoglycoside monotherapy.

#### Publication bias and sensitivity analysis

Publication bias was assessed using an adjusted funnel plot (Supplementary Figure S4), which revealed a predominantly symmetrical distribution of studies clustered near the plot’s center, reflecting moderate sample sizes and minimal bias. However, the presence of several outliers beyond the 95% confidence limits suggested potential heterogeneity among the included studies. The combined results remained consistent with the original findings even when any single study was omitted during the sensitivity analysis, indicating that the pooled results were robust (Supplementary Table S3).

## Discussion

To our knowledge, this is the first network meta-analysis systematically evaluating antimicrobial efficacy against BSI caused by KPC-Ent. We systematically evaluated the comparative effectiveness of ten antibiotic regimens for the treatment of bloodstream infections (BSI) caused by KPC-producing Klebsiella pneumoniae (KPC-Ent). A total of 19 studies spanning a 14-year period were included, comprising 18 retrospective cohort studies and one prospective cross-sectional investigation. The overall quality of the included studies was high, with 18 studies rated as low risk of bias and one as moderate risk. Regarding all-cause mortality, the consistency model was selected, and ten treatment strategies were evaluated. SUCRA analysis ranked MEM_Mono lowest (84.0%; PrBest 64.0%), followed by AMG_Mono (84.0%; 17.7%) and CBP_Comb (72.9%; 7.8%). Inconsistency analysis indicated no significant disagreement between direct and indirect evidence. Subgroup analyses by mortality timepoints showed AMG_Mono ranked highest for 28-day mortality (SUCRA 91.3%), while MEM_Mono still ranked highest for 30-day mortality (83.2%). Publication bias assessment revealed a generally symmetrical funnel plot, suggesting a low degree of heterogeneity. Sensitivity analysis confirmed the robustness of the findings, as no single study altered the overall results.

Moreover, the findings demonstrate that combination antibiotic regimens are associated with significantly reduced mortality rates compared to monotherapy, aligning with existing evidence from observational studies (22, 23). A previous analysis revealed that carbapenem-containing combination regimens were associated with significantly improved survival outcomes when the meropenem minimum inhibitory concentration (MIC) was ≤ 8 mg/L (22, 23). However, this finding remains controversial, as results from a previous study showed that the survival benefit of combination therapy was statistically significant only in high-risk subgroups, particularly patients with septic shock or advanced comorbidities (23). Another study found that combination regimens showed unadjusted survival improvements exclusively for pulmonary infection cases presenting with high disease severity (APACHE III > 15) (55). Furthermore, Gutiérrez-Gutiérrez’s study suggested that combination antimicrobial therapy demonstrated significant survival benefits exclusively in high-risk patients stratified by the INCREMENT-CPE mortality scoring system (56). The potential reasons for these divergent treatment effects may include the heterogeneity in the types of agents used, dosages, or baseline characteristics of patients.

Pounaras et al. demonstrated that KPC-Ent is sensitive to meropenem but may harbor a subgroup exhibiting drug resistance; therefore, treatment with this antibacterial agent alone may lead to treatment failure (57). A previous systematic review of 432 KPC-Ent BSI patients concluded that combination therapy, especially those containing carbapenems, is superior to COL or TGC alone, suggesting potential additive or even synergistic effects (58). These results suggest that, although meropenem may be the most effective treatment due to synergistic effects and reduced resistance, combination antibiotic regimens should still be considered for better clinical outcomes (59, 60). Based on the SUCRA ranking results of this study, we advise clinicians to consider Carbapenem-based Combinations and Meropenem-based Combinations to mitigate potential drug resistance caused by monotherapy. Furthermore, the underlying adverse effects warrant further investigation. When antibiotics commonly used for *Klebsiella pneumoniae* infection are ineffective, other drugs such as COL and TGC are also administered to treat infected patients (61). COL and TGC remain active against most KPC-Ent strains *in vitro*, and they are currently the preferred treatments for BSI. However, emerging resistance patterns to these antimicrobial agents have been increasingly documented in recent surveillance studies (62). Combinations involving either of the two antibiotics or both are needed to achieve optimal antibiotic effects (22, 40, 44, 48, 63). In 2015, the Food and Drug Administration (FDA) approved CAZ-AVI, a novel β-lactam/β-lactamase inhibitor combination demonstrating potent *in vitro* and clinical activity against KPC-Ent (64), which was proved to be associated with decreased mortality in our study. A recent meta-analysis of observational cohort studies demonstrated superior clinical outcomes with CAZ-AVI versus alternative antimicrobial regimens for KPC-Ent infections (65). Additionally, the prevalence of KPC-Ent in each country is different. High prevalences of carbapenem resistance have been reported in Greece (64.7%) and Italy (29.7%), while low prevalences have been registered in Norway and Sweden (0.1%) (66), which suggested that the geography, relevant country-specific factors, and drug treatment policies may cause this difference. Thus, to ultimately establish the efficacy and safety of these therapeutic regimens against KPC-Ent, meticulously designed RCTs are imperative.

It is known that the combination of COL and TGC is often discontinued due to renal toxicity in clinical practice (67). The standard TGC regimen, as a second-generation tetracycline-class antibacterial agent, regimen consists of a 100 mg loading dose followed by 50 mg every 12 h. However, emerging clinical evidence has raised concerns regarding its therapeutic efficacy, with meta-analyses demonstrating a significant association between TGC use and elevated all-cause mortality (68). This safety concern prompted the FDA to issue a black-box warning in 2010 regarding increased mortality risk. Recent pharmacokinetic-pharmacodynamic analyses suggest that suboptimal drug concentrations at infection sites may contribute to these poor outcomes (69). Consequently, experts have advocated for reevaluation of TGC’s clinical breakpoints to optimize its therapeutic utility. Additionally, CAZ-AVI showed a consistent safety profile with that of ceftazidime monotherapy, including eosinophilia, transient increases in hepatic enzymes, positive Coomb’s test, nausea, and diarrhea based on several phase II and III clinical trials (70, 71). Therefore, physicians are advised to undertake a thorough risk-benefit assessment when considering this intervention.

Based on the findings of this study, we offer several practical implications for the management of KPC-Ent bloodstream infections. First, CAZ_AVI remains a robust treatment option, with demonstrated superiority over carbapenem monotherapy and comparable effectiveness to carbapenem-based combinations. Secondly, conducting more well-designed RCTs and investigating the differential treatment responses across various KPC-Ent strain subtypes (72) is necessary to robustly confirm the efficacy and safety of these regimens against KPC-Ent infections. Lastly, we suggest future studies to perform network meta-analyses that evaluate novel antibiotics (e.g., cefiderocol) alongside established regimens (73) and assess the effectiveness of different therapeutic approaches in high-risk patient subpopulations, particularly those with septic shock (23).

Like other NMAs, this study has several limitations. First, it is based solely on observational studies, with no RCTs included. Despite our efforts to perform subgroup analyses and assess study quality, the inherent risk of confounding factors, such as patient age, underlying health conditions, inpatient departments, geographical location, country-specific regulations, and drug treatment policies, means that the pooled effect estimates should be interpreted as evidence of association rather than causation. Nevertheless, based on the NOS scale, the overall risk of bias in the included studies was low, providing valuable insights into therapeutic strategy against KPC-Ent BSI. Second, data on the safety and resistance of antibiotic regimens for KPC-Ent BSI were not reported by most original studies or could not be synthesized, thus preventing this study from providing evidence on safety. Third, subgroup analyses by a range of effect modifiers, including types of agents used, dosages, or baseline characteristics of patients could not be performed to determine the source of heterogeneity owing to insufficient sample sizes across individual treatment arms, precluding meaningful stratification of the study population. Fourth, the treatment regimens exhibited significant diversity in terms of agent type and dosage. A manual classification was performed based on the original regimens, which could inevitably introduce bias into the overall results. Finally, the limited number of studies evaluating individual antibiotic regimens necessitates cautious interpretation of the findings.

## Data Availability

The original contributions presented in the study are included in the article/Supplementary material, further inquiries can be directed to the corresponding author.
