# Supplementary material for: Effect of antibiotic regimens for bloodstream infections caused by KPC-producing enterobacter: a network meta-analysis
Source: Front Med (Lausanne). 2026 Jun 30;13:1743758. doi: 10.3389/fmed.2026.1743758 (PMC13364693; doi:10.3389/fmed.2026.1743758)
Supplement: Supplementary file 1 [file Data_Sheet_1.pdf]

Table S1. Search strategies for databases

Search strategy of PubMed

NO.	Search Details	Results
#8	(#1 OR #4) AND (#2 OR #5) AND (#3 OR #6) Filter: Humans	1,472
#7	(#1 OR #4) AND (#2 OR #5) AND (#3 OR #6)	2,118
#6	((((((((((((((((Bloodstream Infection) OR (Bloodstream Infections)) OR (Pyemia)) OR (Pyemias)) OR (Pyohemia)) OR (Pyohemias)) OR (Pyaemia)) OR (Pyaemias)) OR (Septicemia)) OR (Septicemias)) OR (Blood Poisoning)) OR (Blood Poisonings)) OR (Severe Sepsis)) OR (abdominal sepsis)) OR (focal sepsis)) OR (intraabdominal sepsis)) OR (sepsis syndrome)) OR (septic disease)) OR (sepsis)	393,407
#5	((((((((((((((Hyalococcus pneumoniae) OR (Klebsiella pneumoniae aerogenes)) OR (Bacillus pneumoniae)) OR (Bacterium pneumoniae crouposae)) OR (Klebsiella rhinoscleromatis)) OR (b. friedlander)) OR (bacterium pneumonie crouposae)) OR (friedlaender bacillus)) OR (friedlander bacillus)) OR (k. pneumoniae)) OR (klebsiella crouposa)) OR (Klebsiella Pn)) OR (klebsiella pneumonia)) OR (pneumobacillus)) OR (Klebsiella pneumoniae) OR (Klebsiella pneumoniae carbapenemase) OR (KPC) OR (Enterobacter hormaechei) OR (Enterobacterales)	52,380
#4	((((((Carbapenem Antibiotics) OR (Carbapenem)) OR (carbapenems antibiotics)) OR (carbapenems)) OR (carbapenem derivative)) OR (Carbapenem resistan)) OR (carbapenems resistan)	33,070
#3	"Sepsis"[Mesh]	155,576
#2	"Klebsiella pneumoniae"[Mesh] OR "Citrobacter freundii"[Mesh]	21,517
#1	"Carbapenems"[Mesh]	17,707

Search strategy of EMBASE

No.	Query	Results
#8	#7 AND 'human'/de	3139
#7	(#1 OR #2) AND (#3 OR #4) AND (#5 OR #6)	3429
#6	'bloodstream infection':ti,ab,kw OR 'bloodstream infections':ti,ab,kw OR 'pyemia':ti,ab,kw OR 'pyemias':ti,ab,kw OR 'pyohemia':ti,ab,kw OR 'pyohemias':ti,ab,kw OR 'pyaemia':ti,ab,kw OR 'pyaemias':ti,ab,kw OR 'septicemia':ti,ab,kw OR 'septicemias':ti,ab,kw OR 'blood poisoning':ti,ab,kw OR 'blood poisonings':ti,ab,kw OR 'severe sepsis':ti,ab,kw OR 'abdominal sepsis':ti,ab,kw OR 'focal sepsis':ti,ab,kw OR 'intraabdominal sepsis':ti,ab,kw OR 'sepsis syndrome':ti,ab,kw OR 'septic disease':ti,ab,kw OR 'sepsis':ti,ab,kw	278293
#5	'sepsis'/exp	419269
#4	'hyalococcus pneumoniae':ti,ab,kw OR 'klebsiella pneumoniae aerogenes':ti,ab,kw OR 'bacillus pneumoniae':ti,ab,kw OR 'bacterium pneumoniae crouposae':ti,ab,kw OR 'klebsiella	63469

	rhinoscleromatis':ti,ab,kw OR 'b. friedlander':ti,ab,kw OR 'bacterium pneumonie crouposae':ti,ab,kw OR 'friedlaender bacillus':ti,ab,kw OR 'friedlander bacillus':ti,ab,kw OR 'k. pneumoniae':ti,ab,kw OR 'klebsiella crouposa':ti,ab,kw OR 'klebsiella pn':ti,ab,kw OR 'klebsiella pneumonia':ti,ab,kw OR 'pneumobacillus':ti,ab,kw OR 'klebsiella pneumoniae':ti,ab,kw OR 'Klebsiella pneumoniae carbapenemase':ti,ab,kw OR 'KPC':ti,ab,kw OR 'Enterobacter hormaechei':ti,ab,kw OR 'Enterobacterales':ti,ab,kw	
#3	'klebsiella pneumoniae'/exp OR 'Citrobacter freundii'/exp	79806
#2	'carbapenem antibiotics':ti,ab,kw OR 'carbapenem':ti,ab,kw OR 'carbapenems antibiotics':ti,ab,kw OR 'carbapenems':ti,ab,kw OR 'carbapenem derivative':ti,ab,kw OR 'carbapenem resistan':ti,ab,kw OR 'carbapenems resistan':ti,ab,kw	35241
#1	'carbapenem derivative'/exp	13834

Search strategy of Cochrane Library

NO.	Search deatiles	Hits
#1	MeSH descriptor: [Carbapenems] explode all trees	941
#2	MeSH descriptor: [Klebsiella pneumoniae] explode all trees	143
#3	MeSH descriptor: [Sepsis] explode all trees	6706
#4	(Carbapenem Antibiotics):ti,ab,kw OR (Carbapenem):ti,ab,kw OR (carbapenems antibiotics):ti,ab,kw OR (carbapenems):ti,ab,kw OR (carbapenem derivative):ti,ab,kw OR (Carbapenem resistan):ti,ab,kw OR (carbapenems resistan):ti,ab,kw	785
#5	(Hyalococcus pneumoniae):ti,ab,kw OR (Klebsiella pneumoniae aerogenes):ti,ab,kw OR (Bacillus pneumoniae):ti,ab,kw OR (Bacterium pneumoniae crouposae):ti,ab,kw OR (Klebsiella rhinoscleromatis):ti,ab,kw OR (b. friedlander):ti,ab,kw OR (bacterium pneumonie crouposae):ti,ab,kw OR (friedlaender bacillus):ti,ab,kw OR (friedlander bacillus):ti,ab,kw OR (k. pneumoniae):ti,ab,kw OR (klebsiella crouposa):ti,ab,kw OR (Klebsiella Pn):ti,ab,kw OR (klebsiella pneumonia):ti,ab,kw OR (pneumobacillus):ti,ab,kw OR (Klebsiella pneumoniae):ti,ab,kw OR 'Klebsiella pneumoniae carbapenemase':ti,ab,kw OR 'KPC':ti,ab,kw OR 'Enterobacter hormaechei':ti,ab,kw OR 'Enterobacterales':ti,ab,kw	1118
#6	(Bloodstream Infection):ti,ab,kw OR (Bloodstream Infections):ti,ab,kw OR (Pyemia):ti,ab,kw OR (Pyemias):ti,ab,kw OR (Pyohemia):ti,ab,kw OR (Pyohemias):ti,ab,kw OR (Pyaemia):ti,ab,kw OR (Pyaemias):ti,ab,kw OR (Septicemia):ti,ab,kw OR (Septicemias):ti,ab,kw OR (Blood Poisoning):ti,ab,kw OR (Blood Poisonings):ti,ab,kw OR (Severe Sepsis):ti,ab,kw OR (abdominal sepsis):ti,ab,kw OR (focal sepsis):ti,ab,kw OR (intraabdominal sepsis):ti,ab,kw OR (sepsis syndrome):ti,ab,kw OR (septic disease):ti,ab,kw OR (sepsis):ti,ab,kw	19005
#7	MeSH descriptor: [Citrobacter freundii] explode all trees	3
#8	(#1 OR #4) AND (#2 OR #5 OR #7) AND (#3 OR #6)	55

Search strategy of web of science

NO.	Search deatiles	Hits
#1	(((((TS=(Carbapenem Antibiotics) OR TS=(Carbapenem)) OR TS=(carbapenems antibiotics)) OR TS=(carbapenems)) OR TS=(carbapenem derivative)) OR TS=(Carbapenem resistan)) OR TS=(carbapenems resistan)	27352
#2	((((((((((((TS=(Hyalococcus pneumoniae) OR TS=(Klebsiella pneumoniae aerogenes)) OR TS=(Bacillus pneumoniae)) OR TS=(Bacterium pneumoniae crouposae)) OR TS=(Klebsiella rhinoscleromatis)) OR TS=(b. friedlander)) OR TS=(bacterium pneumonie crouposae)) OR TS=(friedlaender bacillus)) OR TS=(friedlander bacillus)) OR TS=(k. pneumoniae)) OR TS=(klebsiella crouposa)) OR TS=(Klebsiella Pn)) OR TS=(klebsiella pneumonia)) OR TS=(pneumobacillus)) OR TS=(Klebsiella pneumoniae) OR TS=(Citrobacter freundii) OR TS=(Klebsiella pneumoniae carbapenemase) OR TS=(KPC) OR TS=(Enterobacter hormaechei) OR TS=(Enterobacterales)	85896
#3	((((((((((((((((TS=(Bloodstream Infection) OR TS=(Bloodstream Infections)) OR TS=(Pyemia)) OR TS=(Pyemias)) OR TS=(Pyohemia)) OR TS=(Pyohemias)) OR TS=(Pyaemia)) OR TS=(Pyaemias)) OR TS=(Septicemia)) OR TS=(Septicemias)) OR TS=(Blood Poisoning)) OR TS=(Blood Poisonings)) OR TS=(Severe Sepsis)) OR TS=(abdominal sepsis)) OR TS=(focal sepsis)) OR TS=(intraabdominal sepsis)) OR TS=(sepsis syndrome)) OR TS=(septic disease)) OR TS=(sepsis)	230917
#4	#3 AND #2 AND #1	1622

Table S2 Inconsistency analysis of overall mortality using node-splitting method

Side	Direct		Indirect		Difference		P>|z|	tau
	Coef.	Std.Err.	Coef.	Std.Err.	Coef.	Std.Err.		
B vs C	0.642176	0.5764127	-0.4366357	0.7161026	1.078812	0.935024	0.249	0.5031258
B vs D	1.095758	0.714743	1.510199	0.6245672	-0.4144413	0.9575096	0.665	0.5362519
B vs E	0.7731899	0.6756572	-0.4126216	1.287397	1.185811	1.453927	0.415	0.5038945
B vs G	0.6585513	0.7228851	1.224633	0.5387911	-0.5660815	0.8926978	0.526	0.5400345
B vs H	0.9810896	0.6992434	0.5032089	0.4593478	0.4778807	0.840816	0.57	0.5288335
B vs I	0.6919043	0.4909646	1.368462	0.5924461	-0.6765573	0.7709478	0.38	0.5195776
B vs J	0.7744846	0.7753764	1.217567	0.540156	-0.4430821	0.9408481	0.638	0.5431357
A vs B	0.3680586	0.9979934	-0.8022915	0.6387448	1.17035	1.180287	0.321	0.536182
A vs C	-0.6402351	0.9556925	-0.0214376	0.7337365	-0.6187975	1.218611	0.612	0.5032435
A vs D	1.329502	0.6463625	-0.1620339	0.9751282	1.491536	1.207621	0.217	0.5229893
A vs E	-3.992549	2.230907	0.5659398	0.791829	-4.558489	2.3581	0.053	0.4823784
A vs F	-2.035703	1.467964	0.7505253	1.870497	-2.786229	2.198986	0.205	0.4941433
A vs G	0.5411562	0.4927889	0.6390309	1.736485	-0.0978746	1.799582	0.957	0.5146175
A vs H	0.0404673	0.5032968	0.7494069	0.9528954	-0.7089395	1.032661	0.492	0.5323021
A vs I	0.7077053	0.5010736	-0.7324568	1.11281	1.440162	1.175144	0.22	0.5094513
A vs J	0.6183515	0.4914638	0.1587432	2.611906	0.4596083	2.701288	0.865	0.5114113
C vs D	1.799461	0.8081935	0.6407445	0.6590656	1.158716	1.039824	0.265	0.4970067
C vs G	1.012067	0.7694189	0.6701571	0.6224163	0.3419099	0.9784722	0.727	0.514513
C vs H	0.3046889	0.5166143	0.6763093	0.6991255	-0.3716204	0.8295159	0.654	0.5160272
C vs I	2.074325	0.8588713	0.2896164	0.5060064	1.784709	0.9757643	0.067	0.4493549
C vs J	1.488966	0.7459406	0.4158389	0.6108768	1.073127	0.9405155	0.254	0.501781
D vs E	-0.1973153	1.424955	-0.9626112	0.793245	0.7652959	1.517168	0.614	0.5235629

D vs G	-0.4132943	0.4422743	0.4062609	1.151472	-0.8195552	1.239913	0.509	0.5416146
D vs H	-0.7411343	0.5568445	-0.6206879	0.5689428	-0.1204464	0.7863301	0.878	0.5285231
D vs I	0.0060223	0.487892	-1.193287	0.7683265	1.199309	0.9468609	0.205	0.4745357
D vs J	-0.2355295	0.4858246	-0.3163779	0.8248115	0.0808484	0.9542943	0.932	0.5282347
E vs F	-1.657183	2.25619	-0.7503449	1.69662	-0.9068385	2.861815	0.751	0.513858
E vs G	2.436598	1.431997	0.019099	0.7467244	2.417499	1.557873	0.121	0.4906059
E vs H	-0.2253213	1.327217	0.2261716	0.7457838	-0.4514929	1.429515	0.752	0.5204675
E vs I	0.2805765	1.828489	0.4740376	0.7195593	-0.1934611	1.894293	0.919	0.5152268
E vs J	2.476174	1.784795	0.2738056	0.7289348	2.202368	1.868982	0.239	0.4946415
F vs G	6.140696	2.111312	-0.57152	1.45538	6.712216	2.548004	0.083	0.4809238
F vs H	0.6679692	1.26129	2.778845	1.644742	-2.110876	1.532835	0.168	0.5236178
F vs I	0.2554946	1.651741	2.20056	1.351099	-1.945065	1.71603	0.257	0.5395837
F vs J	3.49743	1.727808	0.4261313	1.4414	3.071298	2.035455	0.131	0.4939137
G vs H	-0.3982232	0.4029725	-0.3175445	0.712815	-0.0806787	0.8139178	0.921	0.5357926
G vs I	0.0885089	0.3901534	-0.8527084	0.9516344	0.9412173	1.032522	0.362	0.5175103
G vs J	0.0598116	0.3621758	-0.4943188	2.412698	0.5541304	2.439614	0.82	0.5125956
H vs I	0.2526611	0.3729955	0.5587412	0.6688944	-0.3060801	0.7611263	0.688	0.5365986
H vs J	0.4469881	0.4062244	0.3646143	0.6989053	0.0823738	0.8033218	0.918	0.5350021
I vs J	-0.1979209	0.394525	1.422542	0.8581265	-1.620463	0.9505124	0.088	0.5024415

A: AMG_Mono, B: CAZ_AVI, C: CBP_Comb, D: CBP_Mono, E: MEM_Comb, F: MEM_Mono, G: Others, H: POL_Comb, I: POL_Mono, J: TGC_Mono

Table S3 Results on sensitivity analyses

All-cause mortality

pro-best	AMG_Mono	CAZ_AVI	CBP_Comb	CBP_Mono	MEM_Comb	MEM_Mono	Others	POL_Comb	POL_Mono	TGC_Mono
omitted-Aslan	16.4	4.2	9.1	0.0	8.6	61.3	0.1	0.2	0.0	0.0
omitted-Caston	24.1	3.1	7.9	0.0	6.4	58.4	0.0	0.1	0.0	0.0
omitted-Crooker	8.1	2.0	4.9	0.0	46.3	48.8	0.0	0.0	0.0	0.0
omitted-Daikos	19.1	4.8	4.0	0.1	7.5	63.7	0.1	0.4	0.2	0.1
omitted-Falcone	15.4	4.0	9.7	0.0	6.4	64.0	0.1	0.3	0.0	0.0
omitted-Fang	25.7	1.5	7.9	0.1	9.3	55.2	0.1	0.2	0.0	0.0
omitted-Gomez-Simmonds	12.9	2.7	8.5	0.0	1.5	71.9	0.0	2.4	0.0	0.0
omitted-Gonzalez-Padilla	19.9	4.9	3.7	0.1	7.3	63.2	0.2	0.3	0.3	0.1
omitted-Huang	16.9	3.6	12.2	0	6.5	60.6	0	0.1	0.0	0.0
omitted-Medeiros	2.5	0.5	1.6	0.0	1.1	94.2	0.0	0.0	0.0	0.0
omitted-Papadimitriou-Olivgeris	18.0	3.7	9.5	0.0	7.0	61.7	0.0	0.1	0.0	0.0
omitted-Qureshi	16.9	4.4	10.1	0.0	6.8	61.6	0.0	0.2	0.0	0.0
omitted-Shields	11.6	3.6	19.8	0.0	5.3	59.3	0.0	0.3	0.0	0.0
omitted-Tumbarello	14.7	12.0	6.4	0.0	5.0	61.8	0.0	0.0	0.0	0.0
Pooled estimate	17.5	3.8	9.1	0.0	6.4	63.0	0.0	0.2	0.0	0.0

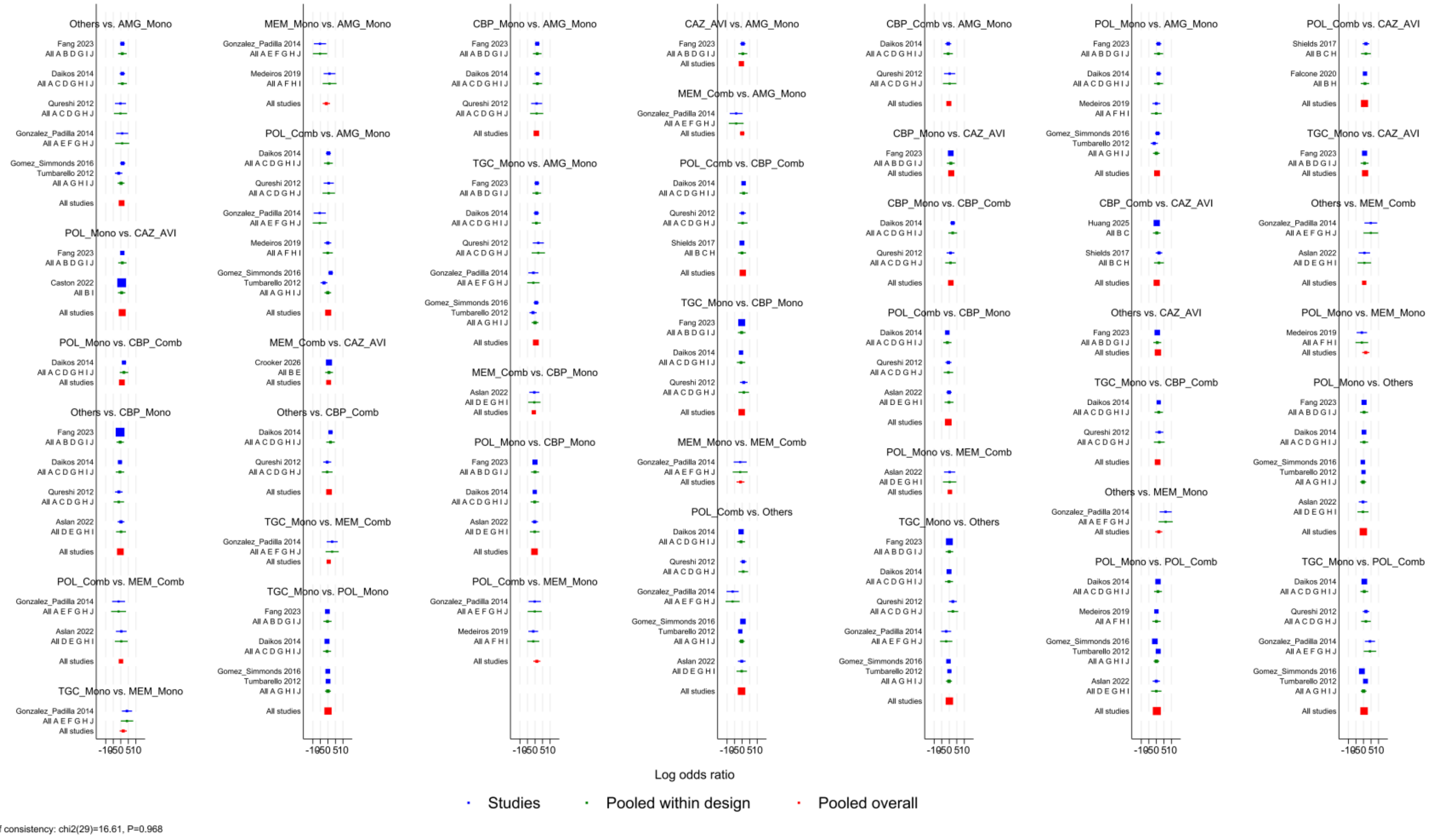

Figure S1 Loop inconsistency of treatment effect on overall mortality

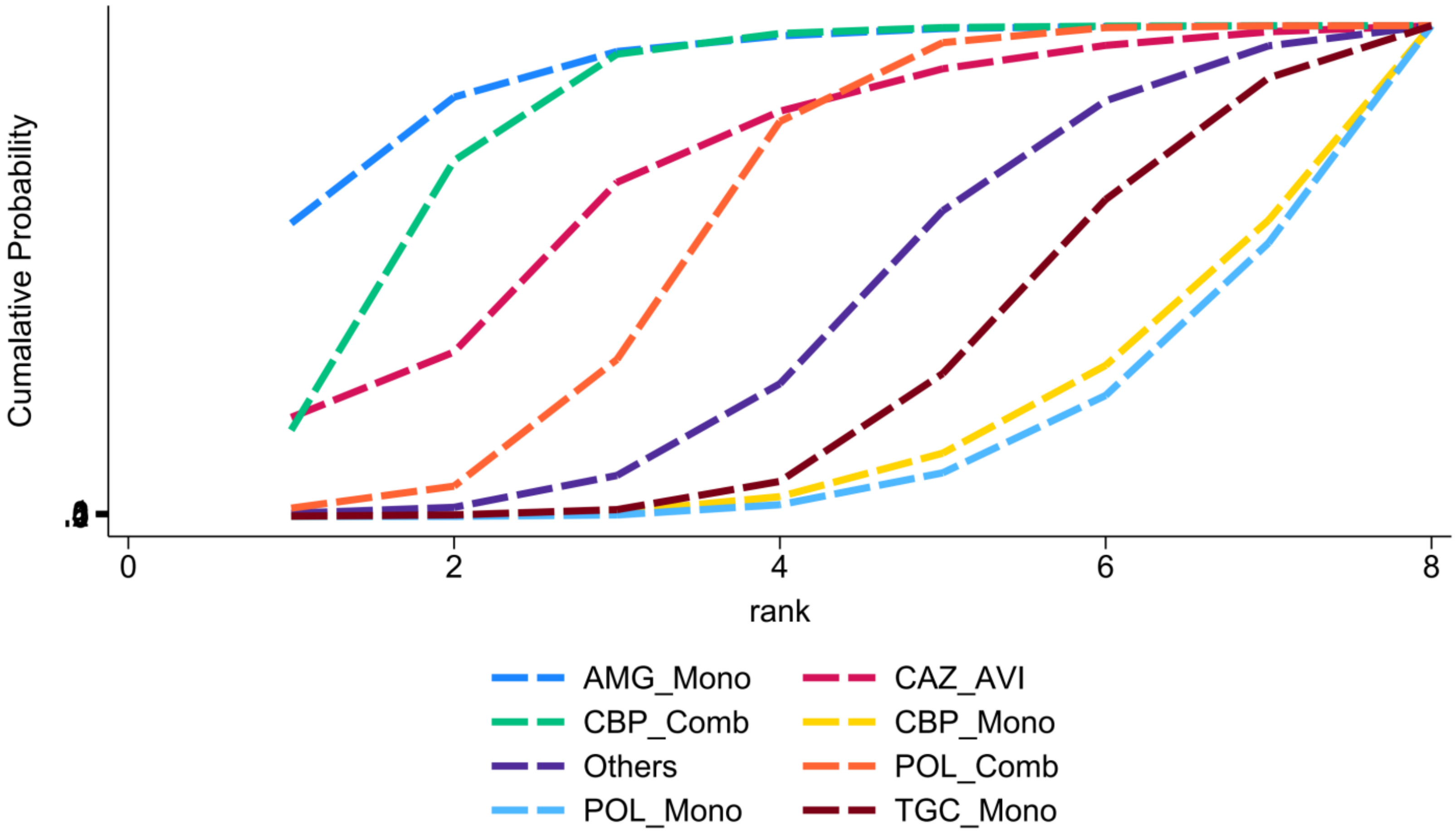

Figure S2 Cumulative probabilities of treatment effect on 28-day mortality

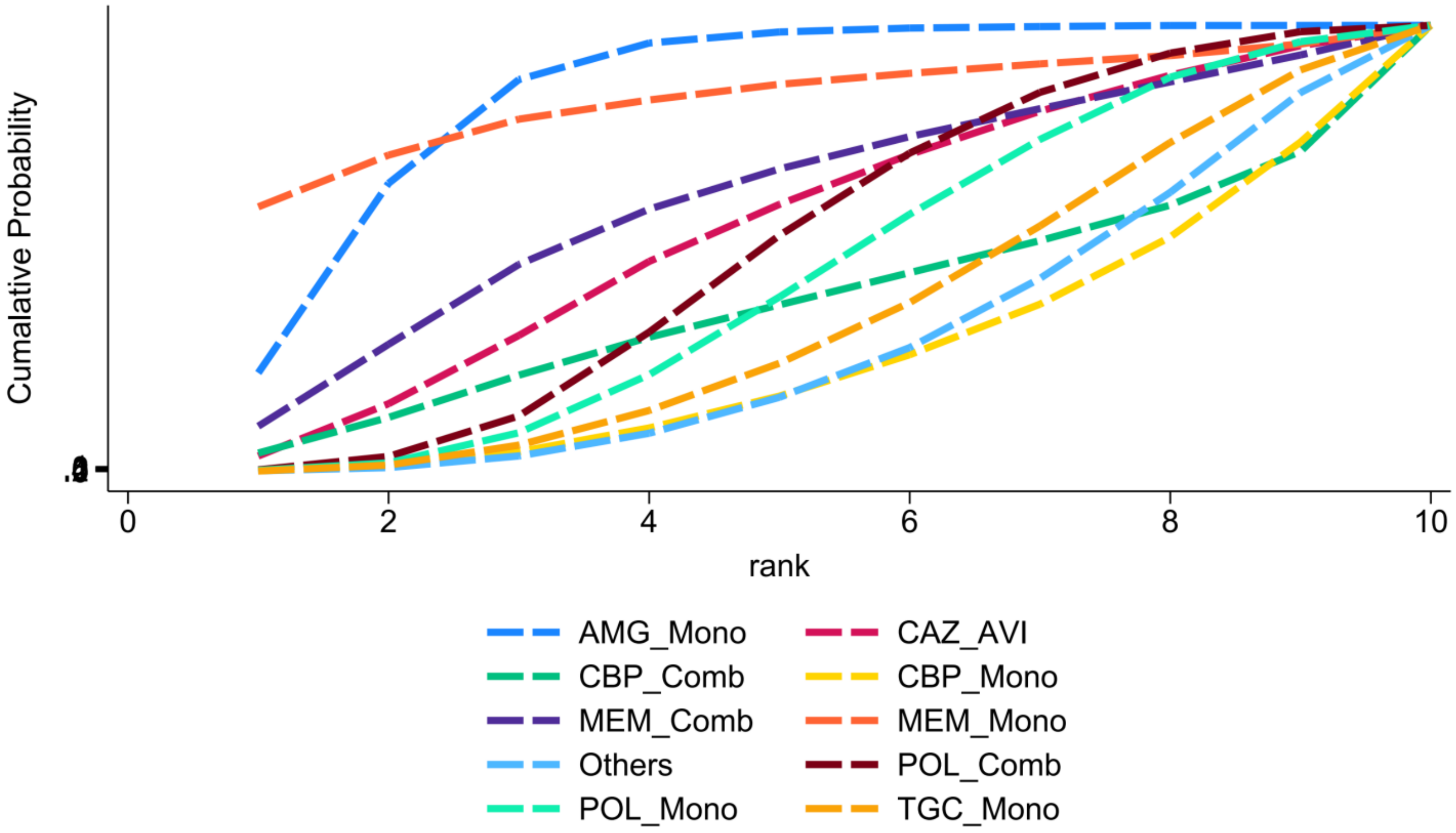

Figure S3 Cumulative probabilities of treatment effect on 30-day mortality

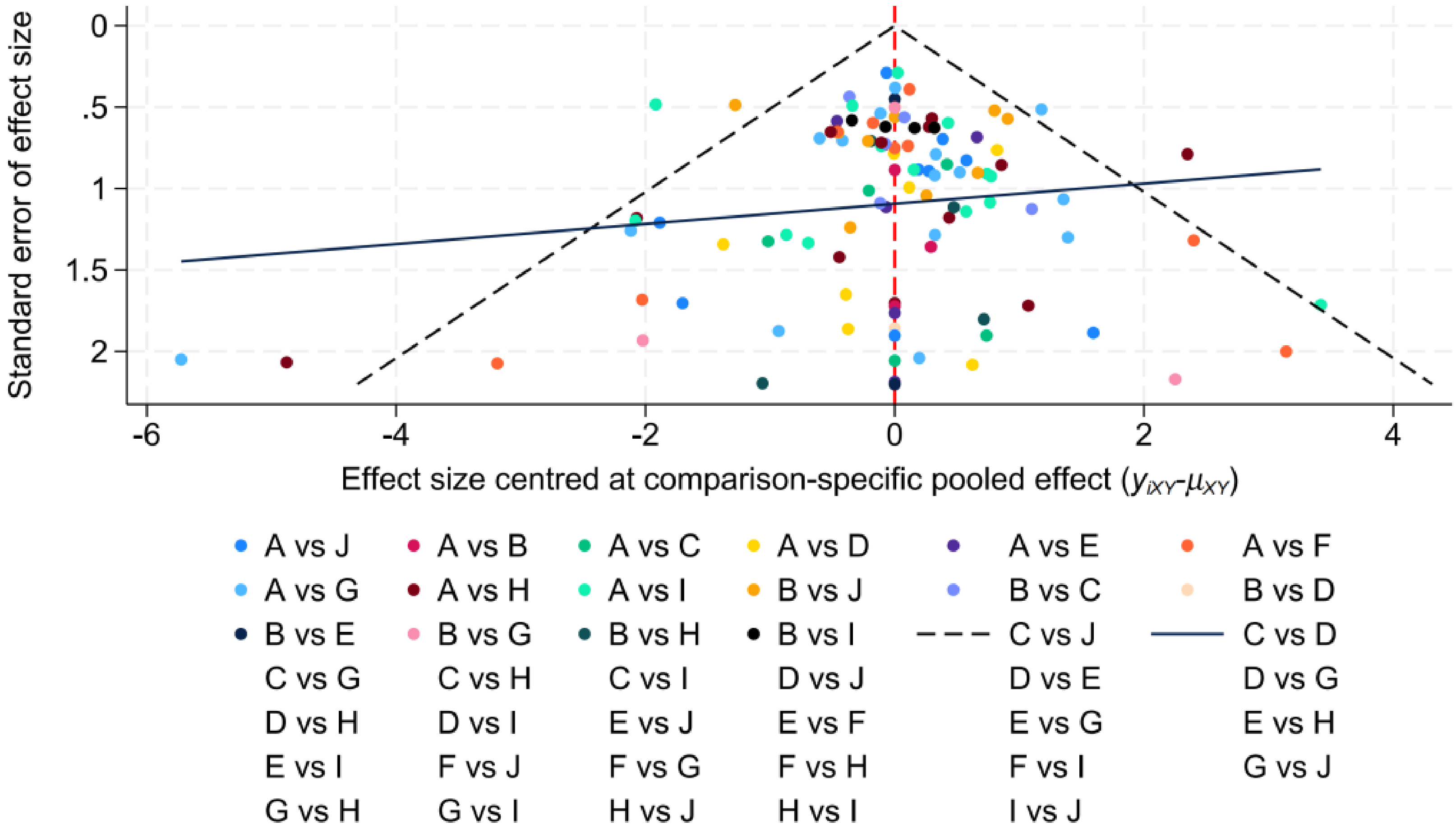

Figure S4 Funnel plot for the analysis of overall mortality
